# A Case of Compound Fracture of the Skull; with Depression of Both Tables, and Laceration of the Membranes and Substance of the Brain

**Published:** 1847-02

**Authors:** T. B. Greenley

**Affiliations:** Westpoint, Kentucky


ART. II.—*A Case of Compound Fracture of the Skull; with depression of both tables, and laceration of the Membranes and Substance of the Brain.* By T. B. GREENLEY, M.D., of Westpoint, Kentucky.

On the 25th August last, Mr. G., a young man, aged about 21 years, of stout make and robust habit, received a severe injury of the cranium, from a blow with an angular-shaped stone. He was deprived of his senses from the time the injury was inflicted. This occurred in the morning directly after an early breakfast. I was sent for immediately, but was not able to reach him before about 4 o'clock in the afternoon. When I arrived I found him lying on the bed, which was very bloody, and the wound still bleeding.

I examined the wound with a probe and found the skull fractured in the region of the parietal eminence; and both tables depressed into the substance of the brain to the depth of an inch. The scalp was cut with angles of the stone so as to form three flaps—the depressed portions of bone being under one of these. In order to remove the depressed pieces of bone it was found necessary to extend one of the angles of the wound, which was done with a scalpel.

After washing off the blood and taking out wads of hair, &c., from the wound, I proceeded to remove the depressed pieces of bone. In the first place I introduced the blunt forceps belonging to a pocket case, but found that I could not insinuate the blades between the fractured pieces, owing to their being carried so evenly before the fulminating body.

The wound being full of blood and still continuing to bleed, compelled me to operate in the dark, or altogether by feeling. After failing to seize the fractured portions with the blunt forceps I was fearful there would be great difficulty in removing them. I however introduced a pair of dressing forceps also belonging to my pocket case, and was able with these owing to their sharp points, to insinuate the point of the blade between two pieces of bone, and force a small piece out. This made room for the introduction of the blunt forceps, before alluded to, with which I soon removed all the remaining pieces—seven or eight in number. After washing the blood out of the wound as well as I could, I found the membranes of the brain, as well as the brain itself, considerably lacerated. I cut off the contused and lacerated portions of membranes and took out several pieces of the substance of the brain, amounting in quantity to about three drachms.

I now brought the flaps of the wound together and closed it with the twisted suture. The hair was then removed from his head, and cloths dipped in cold water applied; venesection at the arm until the system became relaxed; and a purgative dose of medicine administered.

The removal of the depressed portions of bone, together with the loss of blood from the arm, thereby relieving to some extent compression of the brain, appeared to bring him to a partial consciousness of his situation.

It was about 5 o'clock when I got his head dressed and bled him. It will thus be seen that he labored under severe compression of the brain from early in the morning till late in the afternoon, including some nine hours.

Owing to the severity of the case I deemed it necessary that a physician should be with him during the night; and, it being impossible for me to remain, I sent for Drs. Geoghegan and Withers, practitioners of this place, with the hope of getting one of these gentlemen to remain with him. Dr. Geoghegan consented to stay with him all night.

August 26th. I saw the patient to-day about 10 o'clock; his condition about the same as when I left last evening. Had an action on his bowels during the night from the effects of the medicine. Dr. G. informed me that the symptoms continued pretty much the same they were when I left.—Pulse strong and full, but not frequent; tongue slightly furred; pupils nearly natural; action of the kidneys healthy; feet a little inclined to get cold; slight tumefaction at the wound; insensible to objects around him. Abstracted about sixteen ounces of blood from the arm; repeated cathartic medicine; continued application of cloths wrung out of cold water to his head; ordered room to be kept quiet and darkened; and warm rocks, &c., to his feet in case they manifested a disposition to get cold.

27th. Saw the patient about 12 o'clock. No material change in his condition since yesterday. Secretions all appear to be going on properly. Repeated venesection and cathartic medicine. Other treatment continued. Action of the medicine aided by the use of the syringe when necessary.

28th. Saw the patient again to-day about 12 o'clock; condition pretty much the same as on yesterday. Same treatment continued.

29th. Visited him to-day about 5 o'clock in the afternoon. Found him much worse. Convulsions made their appearance last night, and continued to increase in severity and frequency, being very slight at first. While I was there he had a very severe spasm, which lasted nearly an hour. I attributed the convulsions to compression of the brain produced by accumulation of matter in the wound—which had so nearly closed up by the healing process as to obstruct the exit of any matter that might form within. Acting on this supposition I made an incision at the site of the original injury with an abscess lancet, and had a poultice of slippery elm applied. The other treatment pretty much the same as heretofore.

30th. Saw the patient again to-day about 12 o'clock, and was much gratified to find that the convulsions had entirely abated; and there appeared to be some little improvement in his general condition.

The family informed me that the spasms became lighter and less frequent from the time the incision was made the evening before.

The place on his head was mattering very well, having quite a healthy appearance. I was informed by his attendants that a great deal of matter was discharged from the wound during the night. The principal treatment now consisted in keeping his bowels open, and avoiding all exciting causes.

Cold applications continued to his head, and warm ones to his feet when necessary.

His diet has been very light up to this time, in fact scarcely any thing.

31st. To-day the patient appears to be still improving. Is nearly rational: asks for water, &c., whenever he wants it. Can distinguish individuals; comprehend and answer questions. Has some appetite. Same tratment continued. Venesection omitted from yesterday.

Sept. 1st. Still improving. Everything appears to be favorable, and would go to indicate a speedy recovery. The wound presents a healthy aspect, and continues to discharge some pus of a laudable character. Continue poultices of slippery elm to the part, frequently renewed.

3d. To-day I found the patient still improving. He appears to be perfectly rational—conversing rationally on any topic; the only deficiency being a slight stoppage or impediment in his speech. This he says is because he forgets occasionally what word he should use. He is able to go about the house, and out of doors when the weather is pleasant.

6th. To-day I visited my patient for the purpose of leaving directions for his general management, and to dismiss him. He is now in a manner well, with the exception that the wound continues to discharge a small amount of matter, though of quite a healthy character.

I saw him at this place in October, perfectly well in every respect. The wound has healed up; and the stoppage of speech before alluded to, which he labored under a short time, has entirely disappeared. He is now very stout and robust, being much more fleshy than he was previous to receiving the injury.

The recovery of this case may be said to be an extraordinary one. When I first saw him, considering how long it was after the injury was inflicted before he received any surgical or medical aid, the excssive heat of the season, together with the dreadful character of the injury, I unhesitatingly pronounced his recovery impossible. In this opinion I was corroborated by that of Drs. Geoghegan and Withers, both of whom saw him the day the misfortune happened, and the former frequently during his recovery. This opinion was also entertained by every individual who saw him. In fact, I had but little hope of his recovery for a week after he received the injury.

There are not many cases on record, as far as my memory serves me, where recovery took place after the loss of a portion of the brain, although such a fortunate result does occasionally happen.

In the case of this young man I could not help but observe and admire the medicative force of nature; and accordingly have set her down as an able and mighty physician.

December, 1846.

